# Association of weight change patterns in late adolescence with young adult wage differentials: A multilevel longitudinal study

**DOI:** 10.1371/journal.pone.0219123

**Published:** 2019-07-05

**Authors:** Chiao-Yu Huang, Duan-Rung Chen

**Affiliations:** 1 Department of Family Medicine, Renai Branch, Taipei City Hospital, Taipei City, Taiwan; 2 Institute of Health Policy and Management, College of Public Health, National Taiwan University, Taipei City, Taiwan; 3 Institute of Health Behaviors and Community Sciences, College of Public Health, National Taiwan University, Taipei City, Taiwan; Leibniz Institute for Prevention Research and Epidemiology BIPS, GERMANY

## Abstract

**Background:**

Numerous studies have demonstrated that different weight change patterns from adolescence to adulthood may exert different effects on opportunities from which individuals subsequently benefit.

**Objectives:**

This study aimed to investigate the association of weight change patterns from late adolescence to young adulthood with monthly wage in young adulthood for both genders in Taiwan.

**Methods:**

A nationally representative retrospective panel of 3730 young people (1707 men and 2023 women) from the Taiwan Educational Panel Survey (2001–2014) was included. Individuals were divided into four weight-change-pattern categories based on changes in their body mass index at two time points that were 7 years apart, between late adolescence (aged 18–19 years) and young adulthood (aged 25–26 years). These categories were (1) no obesity, (2) obesity reversal, (3) developing obesity, and (4) persistent obesity. Cross-classified, hierarchical linear regression modeling analysis was performed to explore the association of weight change patterns with monthly wage in young adulthood, after adjustment for both individual- and contextual-level variables.

**Results:**

Of the weight-change-pattern categories for both genders, individuals with persistent obesity had the lowest monthly wage. For women, the mean monthly wage decreased progressively for the categories of no obesity, obesity reversal, developing obesity, and persistent obesity (test for difference, P = 0.016; test for trend, P = 0.026). Women with persistent obesity earned 20% less per month than did those who were never obese (P = 0.024), after controlling for individual and contextual factors. For men, no association was found between weight change patterns and monthly wage.

**Conclusion:**

Persistent obesity from late adolescence to young adulthood is associated with low monthly wage in young adulthood in women but not in men. These findings highlight the urgency of addressing persistent obesity early in life, especially for women.

## Introduction

Adolescent obesity is one of the most serious public health problems worldwide [1, 2]. Because adolescence is a critical period when physiological, psychological, and social maturation occurs [3, 4], a change in weight during this period can play a determining role in future outcomes and result in sociopsychological consequences. Adolescents who gain weight and have persistent obesity may experience compromised peer relationships, increased depression risk, and poor quality of life in young adulthood compared with adolescents who have a persistent healthy weight [5–7]. In addition, adolescents who are persistently overweight are more likely to have chronic health problems, to underachieve in education, and to experience unemployment by middle age compared with those with other weight change patterns [8]. From a life-course perspective, different weight change patterns from adolescence to adulthood may affect future life chances in various ways, and this area of investigation has not been fully explored in the Asian context [9, 10].

A more thorough understanding of how obesity’s economic consequences unequally affect men and women is urgently required. Many studies have consistently reported that adult obesity exerts a negative effect on current labor market outcomes, such as wages, particularly for women, because of decreased labor productivity or pure discrimination [11–13]. However, only a few studies have investigated the relationship between the weight change pattern from adolescence to adulthood and future adult wage or income [8, 14]. A British birth cohort of 12,537 participants showed an association of both persistent obesity and obesity reversal after 16 years of age with lower earnings at 23 years of age in women [14]. To date, little evidence demonstrates the effect of different weight change patterns from adolescence to young adulthood with particular focus on Asian populations.

Income is multifactorially influenced by individual factors such as educational attainment [15], mental health status [16], family [17], employment status, and characteristics of respective companies [18–20]. In addition, negative relationships between obesity and socioeconomic attainment are more prominent in higher income areas and occupations requiring more social interactions [21–24]. To adjust for both individual- and contextual-level variables, a multilevel approach was required. In this study, we retrospectively analyzed data separately for each gender from a nationally representative panel survey in Taiwan [25]. We aimed to explore, for both genders, the association between weight change patterns from late adolescence to young adulthood and monthly wages in young adulthood.

## Methods

### Data source

**Fig 1:**
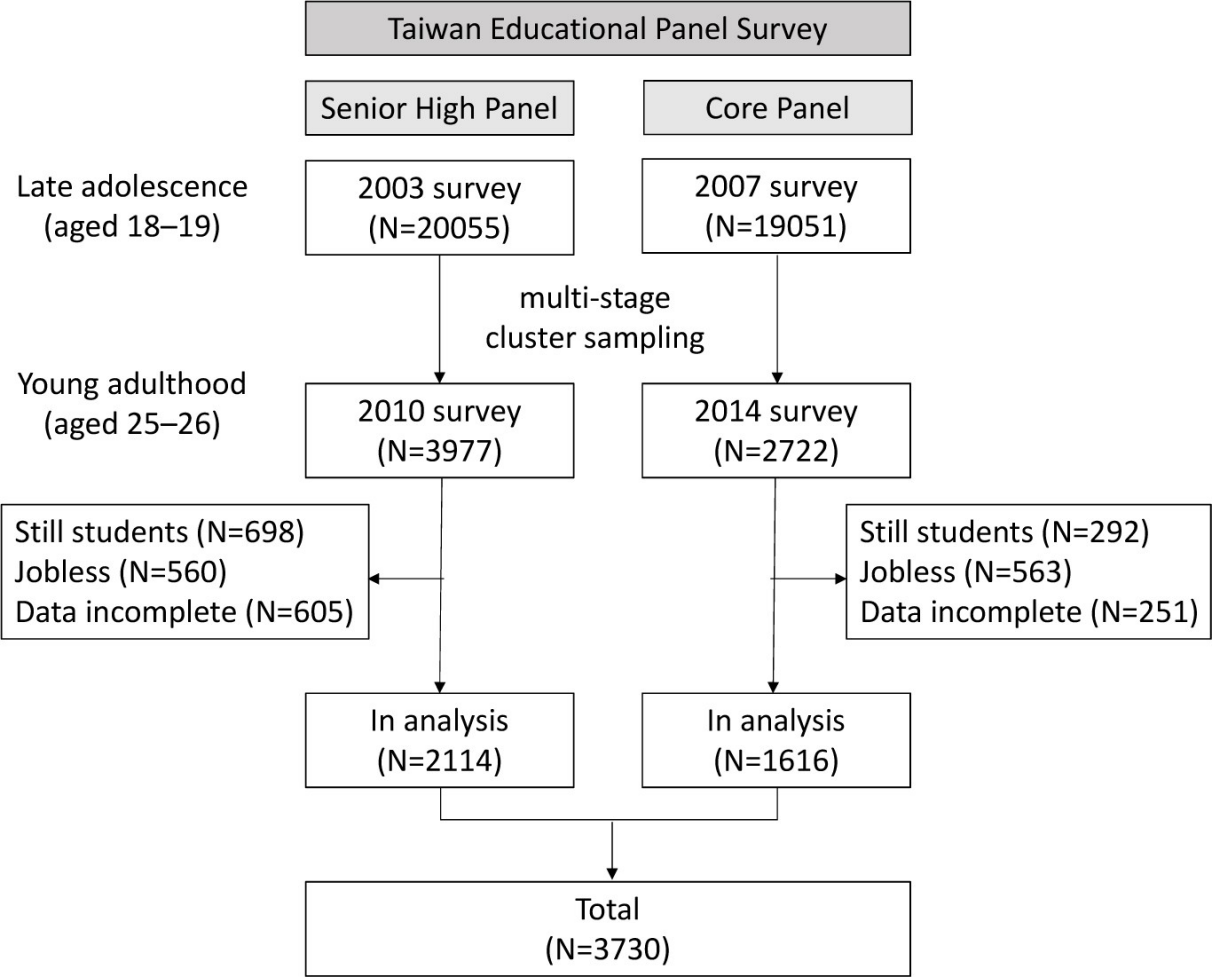
Flowchart of sample selection.

This study used the database from the Taiwan Educational Panel Survey. This is a consecutive panel survey designed to collect longitudinal data from adolescents between 2001 and 2014 in Taiwan [25]. Participants were sampled using a multistage cluster sampling design from the nationwide population [26]. In the first wave of TEPS in 2001, a total of 19,051 senior high school students (born between September 1, 1984, and August 31, 1985, Senior High Panel) and 20,055 junior high school students (born between September 1, 1988, and August 31, 1989; Core Panel) were surveyed. Each panel conducted a follow-up survey in 2003 and 2007, respectively (at the age from 18 to 19 years). For each survey, participating students underwent face-to-face interviews with skilled interviewers who administered structured questionnaires that included demographics-, family-, and education-related questions. A total of 3977 participants from the Senior High Panel and 2722 participants from the Core Panel, sampled using a multistage cluster sampling design from the original panels, participated in follow-up surveys again in 2010 and 2014, respectively (at the age from 25 to 26 years). For each survey, participants underwent face-to-face interviews and reported information on their participation in the labor market during young adulthood. All participants provided informed written consent for data from their records to be used in research. Our study used participants’ data from these two time points (at the age from 18 to 19 years and at the age from 25 to 26 years) to track weight change patterns from late adolescence to young adulthood. Exclusions were made for individuals who were still students, who were jobless at the age of 25 or 26 years, or whose data for relevant variables were incomplete. A total of 3730 participants (2114 participants from the Senior High Panel and 1616 participants from the Core Panel) were included in the analysis. The sample flowchart is depicted in Fig 1. The study protocol was approved by the Institutional Review Board of the Survey Research Data Archive in Academia Sinica, Taiwan. Because the study data contained potentially identifying personal information, such as names of schools and details of residence locations, the data access committee did not allow researchers to download the data (committee contact information: https://srda.sinica.edu.tw/index_en.php). Researchers could only analyze the data online, and all accessed data were fully anonymized.

### Variables

The dependent variable was the monthly wage in young adulthood. The independent variable was the weight change pattern from late adolescence to young adulthood. Control variables included individual-level demographic- and employment-related variables and contextual variables, including location of employment and industry of employment (service or nonservice sectors).

### Definition of terms and measurements

The monthly wage in young adulthood is the self-reported average monthly wage for an individual’s job at the time of data collection (to the nearest NT$1) during the interview (aged 25–26 years).

The weight change pattern is categorized by participants’ obesity status from late adolescence (aged 18–19 years) to young adulthood (aged 25–26 years). Interviewers recorded the self-reported weight (to the nearest 1 kg) and height (to the nearest 0.1 cm) of participants in each survey. Body mass index (BMI) was calculated as weight (kg) divided by height squared (m^2^) and rounded to one decimal place. The World Health Organization (WHO) expert consultation concluded that the proportion of Asian people with a high risk of diabetes and cardiovascular disease is substantial even below the existing WHO BMI cut off point for obesity (BMI ≥30 kg/m^2^) [27]. The Department of Health in Taiwan has therefore officially defined obesity as a BMI of ≥27 kg/m^2^ for Taiwanese people aged 18 years or more. Individuals in our study were classified as obese (BMI ≥ 27 kg/m^2^) accordingly. We grouped individuals into four different weight-change-pattern categories: (1) no obesity (nonobese to nonobese), (2) obesity reversal (obese to nonobese), (3) developing obesity (nonobese to obese), and (4) persistent obesity (obese to obese).

Control variables were constructed using information collected in young adulthood (aged 25–26 years). Individual-level demographic variables included educational level, type of graduation school (private, public, or overseas), parents’ educational level, and depressive symptoms (according to the question “Did you often feel depressed in the past 1 week: yes or no?”). Individual-level employment-related variables comprised job acquisition through social capital (job acquisition through social capital versus job acquisition through other means), contingency of work (typical versus atypical employment), company size (large versus small), working hours, and wage for the first job. Study participants who reported that they received assistance in finding a job from their relatives, friends, teachers, or school contacts were classified as benefiting from job acquisition through social capital; otherwise, they were classified as not benefiting from social capital. Those who had full-time, regular, and open-ended employment were classified as having typical employment; those who had part-time work, temporary work, fixed-term work, casual, or seasonal work or who were self-employed, independent workers, or homeworkers were classified as being in atypical employment. Study participants who worked in companies employing over 300 workers were classified as working in a large company; otherwise, they were classified as working in a small company. To consider the effect of labor supply on wage, working hours, defined as the average amount of time per week spent doing the job held at the time of data collection (to the nearest hour), were included for analysis. The participants’ wage for the first job was defined as the self-reported average monthly wage for their first job when entering the labor market (to the nearest NT$1).

Contextual variables included location of employment and industry type. According to one epidemiologic study, townships in Taiwan can be classified into seven socioeconomic categories (clusters 1 to 7) according to urbanization stratification [28]. Participants in our study were classified as working in an urban area (townships in clusters 1 and 2) or a nonurban area (townships in clusters 3–7). For industry type, participants’ jobs at the time of data collection were classified as being in either a service sector (occupations requiring a high level of social interaction) or a nonservice sector.

### Statistical analysis

Data are summarized as means and standard deviations for continuous variables and as percentages for categorical variables. Differences in monthly wages in young adulthood between study participants with different weight change patterns and between different participant groups categorized according to individual or contextual variables were analyzed using ANOVA and Student's *t*-test for both genders. The Pearson correlation was used to analyze the association of monthly wage for the job held at the time of data collection with working hours and with the participant’s wage for the first job. Data management and statistical analyses were performed using SPSS 11.0 software (SPSS; Chicago, IL). P ≤ 0.05 was considered to be statistically significant.

**Fig 2:**
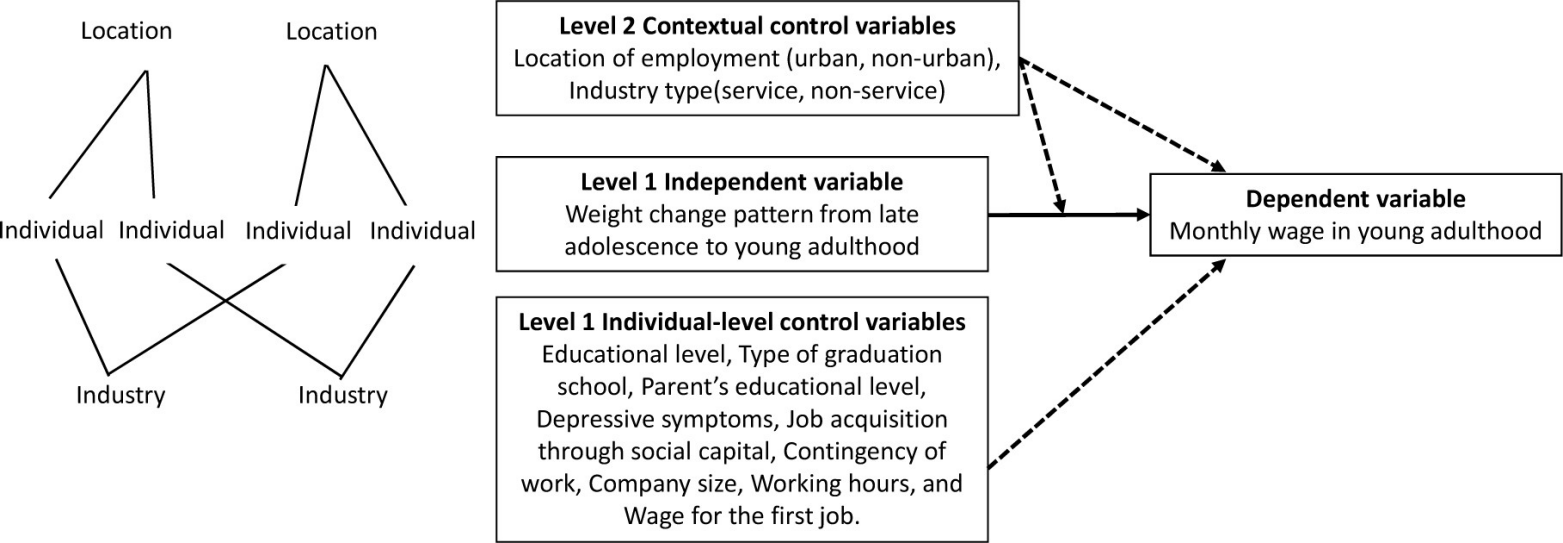
Cross-classified hierarchical structure of analysis.

Data in our study exhibited a cross-classified hierarchical structure, in which a lower level (individual or level 1) factor can independently and simultaneously combine with two non-nested upper level (contextual or level 2) factors (location of employment and industry type) (Fig 2). Thus, we employed cross-classified, hierarchical linear regression modeling to investigate the relationships of monthly wage in young adulthood with weight change patterns and other variables [29]. First, a null model was constructed to assess whether, in our study, monthly wage in young adulthood varied between different contextual settings. The intraclass correlation coefficient (ICC) was computed using the linear threshold model method for generalized hierarchical linear regressions to assess the appropriateness of employing a multilevel model [29]. Next, for the random coefficient model, we introduced several individual variables into the model, and we aimed to investigate the effects of weight change pattern on monthly wage. Finally, the contextual variable was added to the model along with the independent variable, weight change patterns, and other individual variables. Possible cross-level interactions were examined (the association between weight change patterns and wage may be moderated by contextual variables such as employment location and service sector). Both the monthly wage for the job held at the time of data collection (dependent variable) and wage for the first job (independent variable) were log-transformed in the analysis. We could interpret the coefficients of different weight change patterns (dummy variables) as percent changes by using semilogarithmic regression models [30]. Data were analyzed using the statistical program Hierarchical Linear Model (HLM). P ≤ 0.05 was considered to be statistically significant.

## Results

A total of 3730 individuals (1707 men and 2023 women) were included in the analysis. The mean age of participants was 25.4 ± 0.51 years. Table 1 provides the descriptive statistics for men and women. Overall, 159 (9.3%) of men and 87 (4.3%) of women were obese in late adolescence, and 222 (13%) of men and 109 (5.4%) of women were obese in young adulthood. Approximately 65% of those who were obese in late adolescence were also obese in young adulthood; that is, 103 (6.0%) of men and 57 (2.8%) of women had persistent obesity. Among those with persistent obesity in the study, 59 (57%) of men and 34 (60%) of women also showed an increase in the BMI from late adolescence to young adulthood. Furthermore, we examined participants who were still students in young adulthood for sub-analysis. Of these participants (737 after exclusion of missing data, 425 men and 312 women), 625 (84.8%) had never been obese, 58 (7.9%) had obesity reversal, 22 (3.0%) had developing obesity, and 32 (4.2%) had persistent obesity. The distribution was not significantly different from that of our study sample (χ^2^ = 12, P = 0.213).

**Table 1:** Descriptive statistics of study participants (N = 3730).

	Men (N = 1707)			Women (N = 2023)		
	N(%)	Monthly wage*	P	N(%)	Monthly wage*	P
**Level 1 Individual level**						
Weight change patterns			0.236			0.016
No obesity	1429(83.7%)	32023 ± 13750		1874(93%)	30613 ± 12852	
Obesity reversal	56(3.3%)	33193 ± 9772		30(1.5%)	28570 ± 11276	
Developing obesity	119(7.0%)	31699 ± 15284		52(2.6%)	27750 ± 10834	
Persistent obesity	103(6.0%)	29362 ± 10225		57(2.8%)	25960 ± 8226	
Educational level			0.806			0.109
High school	120(7.0%)	31584 ± 10603		76(3.8%)	28089 ± 13900	
College or above	1587(93%)	31900 ± 13772		1947(96%)	30467 ± 12647	
Graduated from			<0.001			<0.001
Private school	649(38%)	30245 ± 12623		807(40%)	28644 ± 9496	
Public school	620(36%)	34425 14265		644(32%)	32403 ± 13536	
Overseas school	438(26%)	30690 13415		572(28%)	30368 ± 15122	
Depressive symptom			0.396			0.142
Yes	327(19%)	32103 ± 13216		288(14%)	31905 ± 20011	
No	1380(81%)	31305 ± 14988		1735(86%)	30124 ± 11016	
Parent’s educational level			0.155			<0.001
Junior high or below	620(36%)	31133 ± 10840		735(36%)	28747 ± 9310	
High school	609(36%)	31977 ± 14892		706(35%)	29775 ± 10493	
College or above	478(28%)	32718 ± 14904		582(29%)	33169 ± 17511	
Job acquisition			<0.001			0.048
With social capital	514(30%)	29914 ± 16321		555(27%)	29470 ± 14165	
Without social capital	1193(70%)	32724 ± 12108		1468(73%)	30721 ± 12088	
Contingency of work			0.041			<0.001
Atypical employment	177(10%)	29908 ± 23837		139(6.9%)	26570 ± 12387	
Typical employment	1530(90%)	32106 ± 11815		1884(93%)	30659 ± 12681	
Company size			<0.001			<0.001
Small	957(56%)	28795 ± 13494		1315(65%)	27971 ± 9120	
Large	750(44%)	35812 ± 12628		708(35%)	34847 ± 16612	
**Level 2 Contextual level**						
Location of employment			0.820			0.001
Non-urban area	649(38%)	31782 ± 11164		595(30%)	28865 ± 9680	
Urban area	1058(62%)	31936 ± 14861		1428(70%)	31008 ± 13718	
Industry type			0.454			0.871
Service industry	689(40%)	32177 ± 14724		1074(53%)	30335 ± 12145	
Non-service industry	1019(60%)	31676 ± 12736		949(47%)	30427 ± 13306	

*data are presented as the mean ± standard deviation

Table 1 show the average mean difference in monthly wage for different weight change patterns. For both genders, participants with persistent obesity or developing obesity earned lower monthly wages than those who were never obese or who had experienced obesity reversal. The lowest monthly wage was observed for those with persistent obesity for both genders. For men, monthly wage was not significantly different among the four types of the weight change patterns. By contrast, for women, the mean monthly wage decreased progressively for the categories of no obesity, obesity reversal, developing obesity, and persistent obesity (test for difference, P = 0.016; test for trend, P = 0.026). Those who experienced persistent obesity earned significantly less than those who had never been obese (P = 0.038, post-hoc analysis, Bonferroni method).

Table 1 shows differences in mean monthly wage between groups of participants categorized by control and contextual variables. At the individual level, participants who graduated from private schools, acquired jobs without social capital, had typical employment, and worked in large companies had significantly higher monthly wages than their counterparts in both genders. Monthly wage was positively correlated with working hours (correlation coefficient, r = 0.317 for men and r = 0.237 for women, P < 0.001) and wage for the first job (r = 0.331 for men and r = 0.249 for women, P < 0.001). Additionally, women whose parents had higher educational level had a significantly higher mean monthly wage. For both genders, at the contextual level, participants who worked in urban areas had higher mean monthly wages than did those who worked in nonurban areas, but statistical significance was found only for women. Monthly wage was not associated with industry type for either gender.

Tables 2 and 3 present the results of a series of cross-classified, hierarchical linear regression models predicting monthly wage in young adulthood for men and women.

**Table 2:** Cross-classified multilevel model describing an association between predictors and monthly wage among men (N = 1707).

	Model 1	P	Model 2	P
**Fix effect estimates**				
Intercept†	10.3(0.08)	<0.001	9.98(0.24)*	<0.001
**Individual-level**				
Weight patterns				
No obesity			Ref	
Obesity reversal			0.288(0.18)	0.107
Developing obesity			0.058(0.13)	0.667
Persistent obesity			0.040(0.13)	0.766
High Educational level			0.293(0.21)	0.163
Graduation from (Private school as ref)				
Public school			0.344(0.33)	0.304
Overseas school			-0.297(0.33)	0.375
Depressive symptom			0.028(0.07)	0.670
Parent’s educational level (Junior high or below as ref)				
High school			-0.021(0.08)	0.790
College or above			-0.142(0.08)	0.086
Job acquisition with social capital			-0.179(0.07)	0.013
Atypical employment			-0.095(0.14)	0.505
Large company			0.392(0.07)*	<0.001
Working hours			0.010(0.002)*	<0.001
Wage for the first job†			0.073(0.03)*	0.014
**Random effect**				
Location (level 2) variance	0.211	<0.001	0.014	0.224
ICC for location of employment	67%		6.4%	
Industry (level 2) variance	0.022	0.002	0.004	0.163
ICC for industry type	7.0%		1.8%	
Individual (level 1) variance	0.082		0.200	

Fixed effect estimates are presented as parameter estimates (standard error).

† Monthly wage at the time of data collection (dependent variable) and wage for the first job (control variable) were log-transformed.

* Statistical significance, P ≤ 0.05

Abbreviations: ICC = Intraclass correlation coefficient

**Table 3:** Cross-classified multilevel model describing an association between predictors and monthly wage among women (N = 2023).

	Model 1	P	Model 2	P	Model 3	P
**Fix effect estimates**						
Intercept†	10.3(0.03)	<0.001	10.0(0.14)*	<0.001	9.95(0.15)*	<0.001
**Individual-level**						
Weight patterns						
No obesity			Ref		Ref	
Obesity reversal			0.196(0.17)	0.264	0.197(0.17)	0.259
Developing obesity			-0.120(0.14)	0.387	-0.116(0.14)	0.423
Persistent obesity			-0.240(0.10)*	0.024	-0.230(0.10)*	0.029
High Educational level			0.219(0.12)	0.060	0.242(0.12)*	0.039
Graduation from (Private school as ref)						
Public school			0.049(0.05)	0.297	0.053(0.05)	0.253
Overseas school			0.090(0.13)	0.482	0.106(0.13)	0.410
Depressive symptom			-0.081(0.10)	0.417	-0.087(0.10)	0.381
Parent’s educational level (Junior high or below as ref)						
High school			0.044(0.05)	0.381	0.039(0.05)	0.415
College or above			0.103(0.06)	0.070	0.098(0.06)	0.084
Job acquisition with social capital			-0.017(0.05)	0.725	-0.010(0.05)	0.822
Atypical employment			-0.034(0.10)	0.730	-0.052(0.10)	0.598
Large company			0.183(0.04)*	<0.001	0.184(0.04)*	<0.001
Working hours			0.009(0.002)*	<0.001	0.009(0.002)*	<0.001
Wage for the first job†			-0.012(0.01)	0.348	-0.012(0.01)	0.361
**Location-level**						
Urban area					0.063(0.04)	0.151
Service industry					0.031(0.05)	0.502
**Random effect estimate**						
Location (level 2) variance	0.080	<0.001	0.011	0.047	0.013	0.017
ICC for location of employment	74%		14%		16%	
Industry (level 2) variance	0.006	0.019	0.00001	0.349	0.00001	0.249
ICC for industry type	5.6%		0.01%		0.01%	
Individual (level 1) variance	0.022		0.069		0.066	

Fix effect estimates are presented as parameter estimates (standard error).

* Statistical significance, P ≤ 0.05

† Monthly wage at the time of data collection (dependent variable) and wage for the first job (control variable) were log-transformed.

Abbreviations: ICC = Intraclass correlation coefficient

For men, as shown in Table 2, Model 1 (the null model) indicated that a considerable amount of variability was attributable to contextual differences (ICC was 67% for inter-location variance and 7% for inter-industry variance). As shown in Table 2, Model 2, when individual variables were added to the model, no significant association was found between weight change patterns and monthly wage for men. However, the mean monthly wage was significantly lower for those who acquired jobs benefiting from social capital (B = −0.179, SE = 0.07) but significantly higher for those who worked in large companies (B = 0.392, SE = 0.07), for those with longer working hours (B = 0.010, SE = 0.002), and for those whose wage for the first job was higher (B = 0.073, SE = 0.03) after adjustments. In addition, the ICC value for contextual variables declined significantly in Model 2, suggesting that contextual variations in monthly wage were largely due to observed individual characteristics.

In women, as shown in Table 3, Model 1 (the null model), a considerable amount of variability was attributable to contextual differences (ICC was 74% for inter-location variance and 5.6% for inter-industry variance). As shown in Table 3, Model 2, when individual variables were added to the model, women with persistent obesity earned a 21% lower wage per month than did those who had never been obese (B = −0.240, SE = 0.175, P = 0.024). However, women with obesity reversal or developing obesity did not exhibit significant earning disadvantages compared with those who had never been obese. In addition, the mean monthly wage was significantly higher for those who worked in a large company (B = 0.183, SE = 0.04) and for those with longer working hours (B = 0.009, SE = 0.002) after adjustments. As the ICC remained significant in Model 2, we added contextual variables into the model. As shown in Table 3, Model 3, the negative effect of persistent obesity on monthly wage remained unchanged, women with persistent obesity earned 20% less per month than did those who were never obese after controlling for other individual and contextual factors (B = −0.230, SE = 0.104, P = 0.029). Women with higher educational level (B = 0.242, SE = 0.12), working in large company (B = 0.184, SE = 0.04) and with longer working hours (B = 0.009, SE = 0.002) are significant associated with higher monthly wage after adjustments. Neither the service industry factor nor the urban area factor was significant in the model, and no cross-level interaction was observed.

## Discussion

In this nationally representative panel study, which tracked weight change over 7 years from late adolescence to young adulthood, we found that different weight change patterns exert different effects on the future adult wage for adolescents in Taiwan, especially in women. After adjustment for individual and contextual confounders, women with persistent obesity from late adolescence to young adulthood earned significantly lower wages compared with those who have never been obese. On the contrary, for men, no association was found between weight change patterns and adult wage.

The cumulative disadvantage theory emphasizes that early disadvantages shape and determine trajectories from an early age and affect long-term outcomes [8, 10, 31]. The effects of early life risk factors accumulate over the life course, thereby increasing heterogeneity in later life. Our study adopted a cumulative disadvantage perspective to explain why persistent obesity from late adolescence to young adulthood in women, but not men, is significantly associated with low monthly wage in later life. Gender differences exist in obesity-related issues among Taiwanese children and adolescents [32, 33]. In Taiwan, body shape–related anxiety is prevalent, especially in female adolescents with obesity [33]; fear of obesity and struggling to lose fat are common conditions that affect girls more than boys [32]. Compared with male adolescents, female adolescents with obesity in Taiwan report more negative effects from weight-based bullying or victimization [32, 33]. Given the gender-specific cultural context in Taiwan, we argue that the process of cumulative early life disadvantages in women with persistent obesity can initiate a chain of negative influences. In particular, women with persistent obesity can be subject to negative sociopsychological experiences from being obese in adolescence (i.e., obesity as a determining factor), and those negative experiences may work through a vicious cycle of psychosocial impairments (i.e., negative sociopsychological experiences as determining factors) that lead to unfavorable socioeconomic conditions in adulthood. Our findings are consistent with the results of other studies conducted on different ethnic groups or in different countries, all of which support the gender-specific disparity of obesity-related socioeconomic outcomes for adolescents [14, 34, 35]. A systematic review also suggested that girls with obesity experience greater lifetime income penalties than boys [36].

Current obesity status is associated with low wages because of workplace weight discrimination [11, 12]. Existing theories to explain this include (1) statistical discrimination, through which people with obesity are likely to be assessed negatively on personnel suitability because they are associated with poor productivity and low self-control, and (2) taste-based discrimination from employers against workers with obesity whose workplaces call for extensive interaction with customers [13, 24, 37]. Consistent with these theories, our study participants with current obesity status in young adulthood, namely those with persistent obesity or developing obesity, earned lower monthly wages than those who were never obese and those who had experienced obesity reversal without adjustments for both genders. However, only persistent obesity in women was a significant independent correlate for low wage after multiple adjustments. Taken together, our results indicate that, for women with persistent obesity, not only does their adolescent obesity have a cumulative negative impact on wage in later life but their current obesity status in adulthood also has a direct wage penalty. Similarly, another study demonstrated a direct wage penalty inflicted by current obesity status and an indirect wage penalty inflicted by late-teen obesity in women based on a nationwide longitudinal survey in Korea [22]. Furthermore, a cohort study indicated that American adults categorized as persistently overweight from the age of 19 years have higher odds of receiving welfare or unemployment compensation at the age of 40 years [8].

The persistence of obesity from adolescence to adulthood is a significant consequence of early obesity in all populations of both genders [3]. The likelihood of persistent obesity as well as the severity of obesity increase with age. In our study, approximately 65% of those with obesity in adolescence were also obese in young adulthood. This was consistent with the findings of a cohort study that tracked participants from the age of 14 years and demonstrated that being obese more than once during adolescence significantly increased the odds of adult obesity [38]. Given that persistent obesity from adolescence poses a significant risk of poor socioeconomic outcomes, an effective intervention program for treating adolescent obesity is urgently required.

Much evidence supports the idea that adolescent obesity is negatively associated with educational attainment, especially in females [34, 39]. Our study demonstrated that female adolescents with obesity had poorer educational attainment than their nonobese peers did. A significantly higher proportion of female adolescents with obesity than female adolescents without obesity failed to complete a college degree (9.2% vs. 3.5% of female adolescents without obesity, P = 0.006) or graduated from private schools (51.7% vs. 39.4% of female adolescents without obesity, P = 0.021), which is less esteemed than graduation from public schools or overseas school in the Taiwanese education system. Furthermore, only 9.2% of female participants with persistent obesity were still students in young adulthood (namely, those who entered postgraduate programs), which is the lowest proportion among all female participants with different weight change patterns. Because educational attainment is widely accepted as a major determinant of wage [15], a previous study assumed that the inverse relationship between adolescent obesity and future wage was mediated by the effects of educational attainment [22]. However, the multivariate cross-classified multilevel models in our study revealed that, for women, both persistent obesity and low educational level are significant independent correlates for low wages after adjustment for both individual- and contextual-level variables.

Some other studies [21, 40], including one study of a Chinese population [23], have suggested that the wage penalty from the current obesity status was more severe for occupations requiring more social interaction, possibly due to taste-based discrimination for women. However, we did not find such interaction of weight change patterns and adult wages with occupation types in the cross-classified multilevel model. We further separated industry into service- and non–service-oriented sectors and examined the association between weight change patterns and wages only for women. The results indicated that, in service-oriented sectors (N = 1059), women with persistent obesity had 13% lower wages than those who had never been obese (P = 0.024); in nonservice sectors (N = 934), women with persistent obesity also earned 10% lower wages than those who had never been obese, although only approaching statistical significance (P = 0.077). We speculate that apart from probable consumer/taste-based discrimination experienced by obese females in service sectors, women with persistent obesity experience cumulative discrimination from late adolescence, which may be why wage penalties for persistent obesity exist both in service and nonservice sectors. Nevertheless, a relatively small sample size limits the extent to which further conclusions may be drawn. Future studies must be conducted to investigate mechanisms underlying respective relationships.

This study is subject to some limitations. First, the validity of the results are debatable. On one hand, the relatively small sample sizes may limit the statistical power. For example, the cross-classified multilevel model for women (Table 3) comes close to indicating that women with obesity reversal earned a higher wage per month and had a better socioeconomic outcome than did those who had never been obese (B = −0.197, SE = 0.17, P = 0.26). However, this nonsignificant result is controversial. For example, a US study showed that those who reached a healthy weight from late adolescence to adulthood still had significantly worse quality of life than those who had a persistent healthy weight [5], whereas another US study indicated that those who had obesity reversal from adolescence to young adulthood had nonsignificantly lower odds of functional disability than those with a persistent health weight [6]. Both these studies did not offer any possible explanations for their findings, and further studies are necessary to examine the effects of obesity reversal. On the other hand, because we extracted and categorized the variables from an existing panel survey, some necessary information, such as subjects’ general health condition, sick leave days and seniority in work, which are all associated with wage [23], were unavailable. However, the majority of study participants entered the formal labor market after graduation from college (approximately 22–23 year-of-age in Taiwan), they all had been working at their companies for less than 3 years. In addition, we controlled confounding effects of the participants’ wage for the first job. Therefore, we believe that the effect of seniority may be limited. Second, we did not include those who were still students or who were jobless in young adulthood, which may have limited the generalizability of results. Third, recall bias may have existed because most variables in our study were self-reported. Although Self-reported weight and, height are highly correlated with measured data in adolescents and adults and are accepted as useful if the data are analyzed categorically for previous studies [11, 41, 42], there is still possibility that the bias of self-reported weight and height may move that person into a different category of weight change pattern. Finally, as a retrospective panel study, we could not demonstrate causal relationships. The reverse causal impact of wage on weight change pattern may not be a serious problem because all study participants had no formal job (therefore no wage) in their late adolescence and approximately 95% of participants had been working for less than 3 years. Even though we controlled variables, such as wage for the first job, participants’ and family’s education attainments, as individual fix effect in the regression analysis, we cannot detailedly explained causality as we lack variables, such as person’s determination, willpower, or genetic predisposition. Further studies are warranted to elucidate these issues.

## Conclusion

This study found that persistent obesity, but not developing obesity, from late adolescence to young adulthood is independently associated with low monthly wage in young adulthood in women but not in men. We speculate that such an association among women is due to both the cumulative disadvantage from being obese and weight discrimination in the workplace. Thus, women with persistent obesity potentially experience multiple negative sociopsychological impacts in adolescence as well as carry these negative consequences to adulthood. Our findings highlight the importance of addressing persistent obesity early in life, especially for women, and additional well-designed studies should be conducted to delineate the mechanism behind the effect of weight change pattern on future outcome.
